# Laparoscopic cholecystectomy for cholecystitis using direct gallbladder indocyanine green injection fluorescence cholangiography: A case report

**DOI:** 10.1016/j.amsu.2020.07.057

**Published:** 2020-08-04

**Authors:** Toshikatsu Nitta, Jun Kataoka, Masato Ohta, Yasuhiko Ueda, Sadakatsu Senpuku, Yukiko Kurashima, Tetsunosuke Shimizu, Takashi Ishibashi

**Affiliations:** a Division of Surgery Gastroenterological Center, Medico Shunju Shiroyama Hospital, Osaka, Japan; b Department of Gastroenterological Surgery, Osaka Medical College, Osaka, Japan

**Keywords:** Laparoscopic cholecystectomy, Indocyanine green, Cholangiography

## Abstract

**<Introduction>:**

Laparoscopic cholecystectomy is the treatment of choice for almost all biliary diseases. We present a novel technique using near-infrared fluorescence imaging for laparoscopic cholecystectomy.

**<Case presentation >:**

A 78-year-old woman diagnosed with acute cholecystitis (Grade II) was scheduled for emergency laparoscopy according to Tokyo Guidelines 2018. We performed a direct percutaneous drainage of the gallbladder to grasp the gallbladder itself. Subsequently, indocyanine green was administered into the gallbladder through the same tube, and the cystic and common bile ducts could be easily detected. The postoperative course was good, and the patient was discharged in remission nine days after the surgery.

**<Discussion>:**

Real-time fluorescence cholangiography with indocyanine green is reliable for biliary anatomy visualization before the dissection of the Calot's triangle. Our method of indocyanine green injection into the same drainage catheter does not require pre-preparation and can be simultaneously performed with drainage intraoperatively. This surgical technique is simple, straightforward, and effective and can be useful in intraoperative decision-making, especially during laparoscopic cholecystectomy.

## Highlights

• A novel technique using ICG.
• Real-time fluorescence cholangiography w.
• Intraoperative decision-making.

## 1 Introduction

Laparoscopic cholecystectomy (LC) has been the gold standard for managing benign biliary diseases. The cost of open cholecystectomy (276700 yen) is higher than that of LC (215000 yen) in the Japanese insurance system [1]. Thus, LC is the first-choice approach for almost all biliary diseases in Japan.

However, bile duct injury (BDI) and postoperative benign bile duct stricture are serious complications of cholecystectomy. The incidence of BDI following open cholecystectomy is 0.2%, whereas that following LC is 2–3 times higher at the range of 0.4%–0.6% [[2], [3]]. [[,^3^] Routine intraoperative cholangiography can reduce the incidence of BDI by providing real-time images of biliary structures^4^,^5^; however, it may be challenging to perform.

Indocyanine green (ICG) fluorescence imaging has been a major factor in intraoperative decision-making. Near-infrared fluorescent cholangiography using ICG was recently introduced as a novel method of visualizing the biliary system.

Here, we present a novel technique of using near-infrared ICG fluorescence during LC in a case of acute cholecystitis.

## 2 Presentation of case

**Fig. 1:**
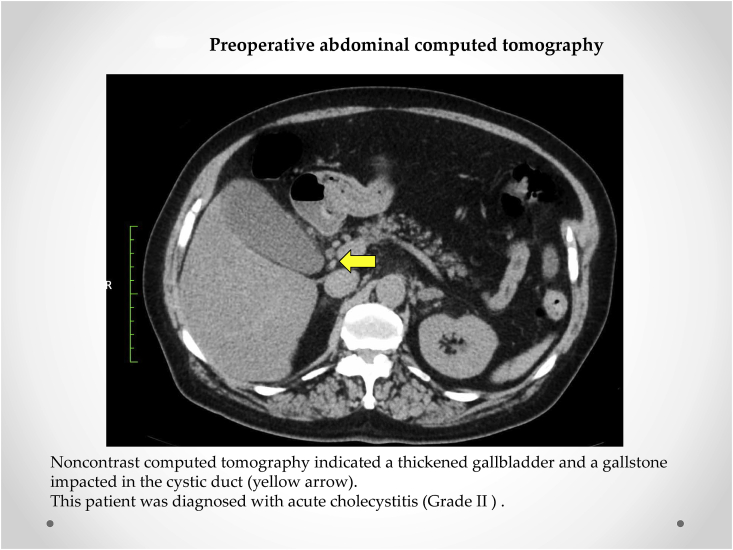
Preoperative abdominal computed tomography. Non-contrast computed tomography indicates a thickened gallbladder and a gallstone impacted in the cystic duct (yellow arrow). This patient was diagnosed with acute cholecystitis (Grade II). . (For interpretation of the references to colour in this figure legend, the reader is referred to the Web version of this article.)

**Fig. 2:**
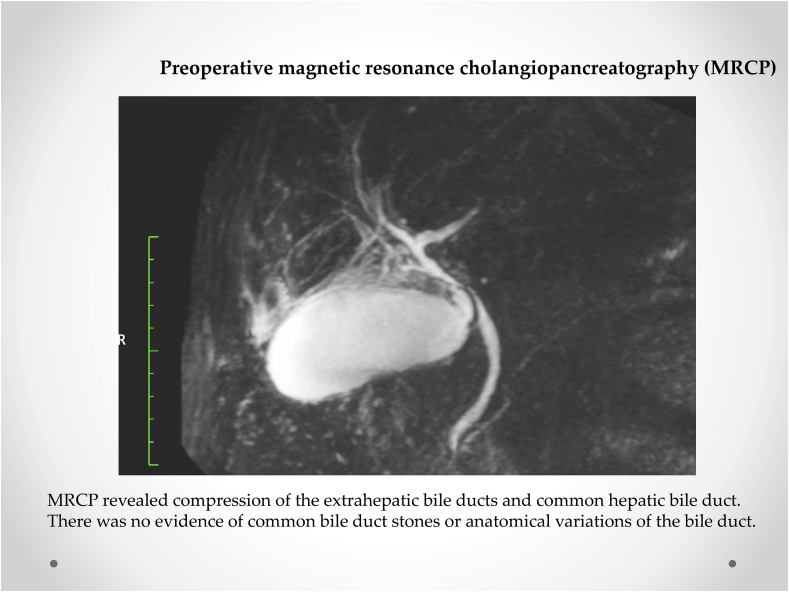
Preoperative magnetic resonance cholangiopancreatography (MRCP). MRCP reveals compression of the extrahepatic bile ducts and common hepatic bile duct. There is no evidence of common bile duct stones or anatomical variations in the bile duct.

A 78-year-old Japanese woman presented to our hospital with pain in the right hypochondrium and high fever (temperature >37.9 °C). She was admitted to our department. On examination, a mass was palpated in the right subcostal region, identified as the gallbladder. The laboratory findings on admission were as follows: white blood cells, 20100/mm^3^ and C-reactive protein, 11.13 mg/dL. Non-contrast computed tomography indicated a thickened gallbladder and a gallstone impacted in the cystic duct (Fig. 1). Magnetic resonance cholangiopancreatography revealed compression of the extrahepatic and common hepatic bile ducts as well as cystic duct obstruction, with no common bile duct stones or anatomical variations in the bile duct (Fig. 2). Thus, the patient was diagnosed with acute cholecystitis (Grade II) and scheduled for emergency laparoscopy according to Tokyo Guidelines (TG18) [6].

**Fig. 3:**
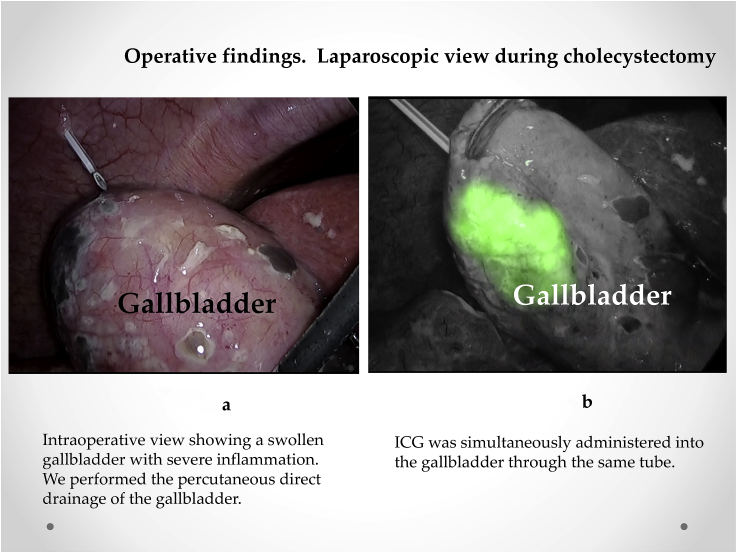
Operative findings. Laparoscopic view during cholecystectomy. a. Intraoperative view showing a swollen gallbladder with severe inflammation. The percutaneous direct drainage of the gallbladder was performed. b. Indocyanine (ICG) is simultaneously administered into the gallbladder through the same tube.

**Fig. 4:**
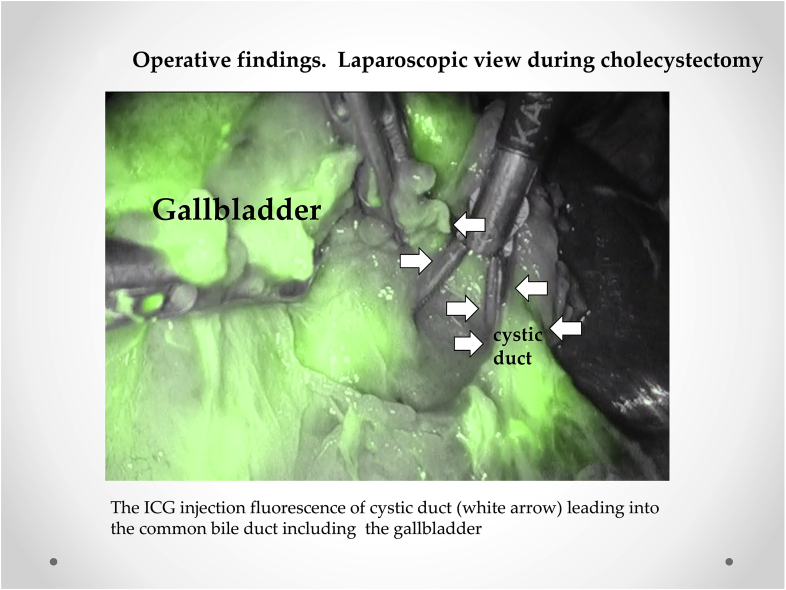
Operative findings. Laparoscopic view during cholecystectomy. The ICG injection fluorescence of the cystic duct (white arrow) leading into the common bile duct including the gallbladder.

Initially, intracorporeal procedures were performed laparoscopically through four trocars. The gallbladder was swollen and tense and was thus difficult to grasp, necessitating direct percutaneous drainage of the gallbladder to grasp it (Fig. 3a). Subsequently, ICG (0.0025 mg/mL of ICG-bile solution) was simultaneously administered into the gallbladder through the same tube (Fig. 3b) and visualized using real-time ICG fluorescence cholangiography. ICG fluorescence cholangiography revealed a swollen gallbladder with inflammation of the Calot's triangle and allowed easy detection of the cystic and common bile ducts (Fig. 4). The cystic artery and duct were skeletonized with blunt dissection, and routine LC for acute cholecystitis was completed.

The patient's postoperative course was good, and she was discharged with remission nine days after the operation. This case report has been reported in line with the SCARE 2018 criteria [7].

## 3 Discussion

LC has become the gold standard in the surgical treatment of acute and chronic cholecystitis since Muhe performed the first LC in 1985 [8]. However, patients undergoing LC should be carefully monitored for BDI. According to the 14th National Survey of Endoscopic Surgery by the Japan Society for Endoscopic Surgery [9], 3397 patients (0.6%) undergoing LC developed BDI, and 234 (0.04%) experienced postoperative bile duct stenosis. In this department, 685 patients underwent cholecystectomy between April 2009 and May 2020, and two cases of BDI (0.29%) and one case of bile duct stenosis (0.14%) were noted. These rates are lower than those reported in other studies [2]. We performed LC in this patient suffering from acute cholecystitis, with intraoperative findings of a difficult gallbladder and Mirizzi syndrome, using our surgical technique [10] and intraoperative cholangiography, which was the only method to intraoperatively delineate bile duct anatomy. Many surgeons [[11], [12], [13]] have reported that routine use of intraoperative cholangiography during LC may reduce the incidence of BDI. Therefore, we performed intraoperative cholangiography to prevent BDI and cannulate the cystic duct.

However, intraoperative cholangiography during cholecystectomy has several disadvantages [14]. First, the most severe complication is increasing the risk of BDI during transcystic tube insertion or cystic duct incision. Second, dissection of the cystic duct itself could be dangerous in the case of a difficult gallbladder. Third, the procedure requires additional medical resources to prevent BDI, such as a tube for cystic duct dilatation and bile duct fiberscope. Fourth, intraoperative cholangiography requires X-ray equipment, which increases the exposure of the patients and surgical staff to radiation. Finally, intraoperative cholangiography increases the operation time [[15], [16]]. [[,^16^].

ICG fluorescence imaging has emerged as a major factor contributing to intraoperative decision-making. Its use enables surgeons to obtain a real-time pathway, and intraoperative ICG injection has shown no side effects [17]. ICG fluorescence cholangiography allows real-time intraoperative visualization of the biliary structures [18], and it is a reliable technique for biliary anatomy before the Calot's triangle dissection.

ICG can be injected using the indirect method, i.e., intravascular ICG (2.5 mg/mL) or the direct method, i.e., transcatheter ICG injection into the bile duct, including the gallbladder (0.025 mg/mL) [14,19]. The direct method was used in this surgical technique, enabling us to perform this as many times as needed, unlike intravascular injection. The direct method equally allows the immediate visualization of the biliary anatomy. We could have injected ICG directly into the percutaneous transhepatic gallbladder drainage (PTGBD) catheter and endoscopic nasobiliary drainage (ENBD) catheter; however, these therapies would have required us to prepare for PTBGD and ENBD before the operation. Using this method, preoperative preparations, such as PTGBD and ENBD, are not necessary, and only the direct injection of ICG during the operation is required.

This technique could be valuable for safe dissection, facilitating a better visualization of the bile ducts while reducing the learning curve. Simultaneously, this method of ICG injection into the drainage catheter is simple, easy to perform, and saves time. In the future, we intend to describe a novel technique of directly injecting ICG into the gallbladder to better visualize the biliary anatomy.

ICG fluorescence cholangiography has been used in laparoscopic surgery, providing surgeons with valuable information [17,20]. Moreover, the imaging device provides direct visualization of the effects of ICG injection during cholecystectomy.

## 4 Conclusion

This surgical technique is simple and effective, and it contributes to intraoperative decision-making, especially during LC.

## Author contribution

Please specify the contribution of each author to the paper, e.g. study concept or design, data collection, data analysis or interpretation, writing the paper, others, who have contributed in other ways, should be listed as contributors.

Toshikatsu Nitta is first Author. Takashi Ishibashi is my supervisor and he checked my paper. Jun Kataoka, Masato Ohta,Yasuhiko Ueda, Sadakatsu Senpuku, Yukiko Kurashima Takahashi Ishibashi, They work under my department of SHIROYAMA HOSPITAL. And they engaged in each operations together.

## Registration of research studies

In accordance with the Declaration of Helsinki 2013, all research involving human participants has to be registered in a publicly accessible database. Please enter the name of the registry and the unique identifying number (UIN) of your study.

You can register any type of research at http://www.researchregistry.com to obtain your UIN if you have not already registered. This is mandatory for human studies only. Trials and certain observational research can also be registered elsewhere such as: ClinicalTrials.gov or ISRCTN or numerous other registries.

Name of the registry: Laparoscopic cholecystectomy for cholecystitis using direct
galllbladder indocyanine green injection fluorescence cholangiography
Unique identifying number or registration ID: researchregistry5805

## Guarantor

The Guarantor is the one or more people who accept full responsibility for the work and/or the conduct of the study, had access to the data, and controlled the decision to publish

My supervisor Takashi Ishibashi is the Gurantor for my publication.

## Funding

The authors did not receive any financial support for this article.

## Ethical approval

All procedures performed in this study were reviewed by the appropriate ethics committee and complied with the principles laid down in the Declaration of Helsinki.

Provenance and peer review.

Not commissioned, externally peer reviewed.

## Declaration of competing interest

The authors have no conflicts of interest to declare.
